# Match loads of university rugby union players between the 2016 and 2018 Varsity Cup competitions

**DOI:** 10.17159/2078-516X/2020/v32i1a7949

**Published:** 2020-01-01

**Authors:** G Gordon, H Morris-Eyton, A Kubayi

**Affiliations:** 1 Department of Sport and Movement Studies, University of Johannesburg, Doornfontein Campus, South Africa; 2 Department of Sport, Rehabilitation and Dental Sciences, Faculty of Science, Tshwane University of Technology, Pretoria, South Africa

**Keywords:** rugby union, match loads, physical demands, position

## Abstract

**Background:**

Rugby union is a popular and continuously growing sport globally. With the advance of technology, practices have been implemented to quantify the match running demands of rugby union players. The aim of this study was to analyse the match loads of rugby union players between the 2016 and 2018 Varsity Cup competitions.

**Methods:**

The sample consisted of 562 match observations of male university rugby union players competing in the Varsity Cup tournaments.

**Results:**

The backline players ran significantly longer total distances (5105 m; *p* = 0.001; ES = 0.49); have greater high-speed running (496 m; *p* = 0.001; ES = 1.03), very high-speed running (260 m; *p* = 0.001; ES = 1.50) and sprint distances (117 m; *p* = 0.001; ES = 1.32) than forward players. Backline players also accumulated a high number of metres per minute (238 ± 94; *p* = 0.001; ES = 0.46), total Player Load (488 ± 203; *p* = 0.001; ES = 0.31), RHIE (9 ± 8; *p* = 0.001; ES = 0.75) and number of accelerations (4 ± 5; *p* = 0.001; ES = 0.49).

**Conclusion:**

These findings may assist coaches to develop player position specific training programmes to meet the physical demands of rugby.

Rugby union has become one of the most exciting and a continuously growing sport around the world.^[1]^ In rugby union, players experience an extensive amount of high-intensity running, with bouts of low intensities which occur throughout matches.^[2]^ The game of rugby requires players to be well-conditioned with respect to endurance, speed, agility, power, flexibility and game-specific skills.^[3,4]^ The use of microtechnology, such as Global Positioning System (GPS) devices, has enabled practitioners to quantify the match loads of rugby union players.^[5,6]^ The knowledge gained from this technology allows for detailed position speciﬁc movement proﬁles that could help facilitate optimal player training programmes in match-play preparation. ^[5,6,7]^

The characteristics of the in-game demands have been explored generally between the forward and backline players in rugby union.^[7]^ Total distance has been reported to be dependent on playing position, with backline players covering greater distance (6471 m) than forwards (5853 m) during matches.^[8]^ In a related study, a detailed analysis of physical demands among professional rugby union players at university level in England was carried out by Read et al. ^[9]^. Findings showed that forwards and backline players covered total distances of 4683 m and 5889 m, respectively. ^[9]^ It was further reported that forwards typically accumulated greater Player Load and Player Load (slow) measures than backs. ^[9]^ The high volume of these metrics could be due to short burst of directional changes associated with backline players during match-play.^[10]^

Differences between positions have also been noted in relation to high-intensity activities during match-play. ^[10]^ A systematic review by Glassbrook et al. ^[11]^ found that backline players covered the greatest relative distance at high speed; however, it was not significantly different to the forwards in the professional rugby league. In contrast, forwards covered significantly less slow-speed distance than the backs. The authors further reported that forwards completed the greatest number of repeated high-intensity efforts (RHIE) over a full match than backs.^[11]^ Previous studies have found that backline players covered a higher number of sprints and accelerations than forward players.^[12,13]^

Despite the physical demands of rugby union players in professional leagues, ^[7, 11]^ there is a paucity of information on university players in South Africa. With the game of rugby union evolving, it is important for coaches to better understand the physical demands of the modern game to implement more specific training programmes.^[4]^ Understanding the physical demands of rugby players during match-play is essential for sports scientists and coaches to develop game-specific conditioning programmes. ^[11, 14]^ The aim of this study was to analyse the match loads of rugby union players between the 2016 and 2018 Varsity Cup competitions.

## Methods

### Research design

This study used a longitudinal retrospective quantitative design utilising secondary data.

### Participants

The sample consisted of 562 match observations of male university rugby union players from 25 matches in the 2016–2018 Varsity Cup tournaments. The players were grouped according to the following playing positions: forwards and backs. Ethical clearance was obtained from the university ethics committee (REC-01-159-2018).

### Data collection

Data were collected by a strength and conditioning coach who used the Catapult Optimeye X4 microtechnology device which was worn by each player in a tight vest during the matches. The microtechnology device has shown levels of accuracy and reliability for distance and speed measurements during intermittent exercise bouts involving high-intensity actions.^[15]^ The distances examined are total distances, as well as distances covered in four key velocity bands. These were moderate speed running (7–16 km/h), high-speed running (16–20 km/h), very high-speed running (20–25 km/h) and sprinting distance (>25 km/h). Player Load expresses arbitrary units of the square root of the sum of the squared instantaneous rates of change in acceleration in each of the three planes of motion and further divided by 100. ^[15]^ The difference between Player Load and Player Load (slow) is that in the latter, the velocities achieved by the players are less than 2 m.s^−1^. ^[9]^ Another metric unit which is widely accepted as a critical variable to consider is RHIE. These efforts take place, with minimal recovery (~6 s), during and after tasks such as tackles, rucks, and accelerations during match-play. ^[14]^ After every match, the data were exported to a Microsoft Excel spreadsheet.

### Statistical analysis

Data were reported as means ± standard deviations. An independent *t*-test was used to compare differences on match loads between back and forward players. Two-way analysis of variance was used to examine the interaction between the year and playing position (backs and forwards) on match running distances of rugby union players. A significance level was set at *p*<0.05. Effect size (ES) was also used to assess the magnitude of the differences in the mean scores of variables. ES values were interpreted as follows: trivial (<0.20); small (0.20–0.59); moderate (0.60–1.19); large (1.20–2.00); and very large (>2.00).^[16]^ All analyses were conducted using the IBM SPSS Version 25.

## Results

Table 1 shows the match running loads of rugby players according to playing position. Backline players showed significantly higher averages than forward players on the following variables: total distance (5105 ± 2150 m; *p* = 0.001; ES = 0.49, small effect), high-speed running (496 ± 258 m; *p* = 0.001; ES = 1.03, moderate effect), very high-speed running (260 ± 136 m; *p* = 0.001; ES = 1.50, large effect), sprinting distance (117 ± 99 m; *p* = 0.001; ES = 1.32, large effect), metre per minute (238 ± 94; *p* = 0.00; ES = 0.46, small effect), total Player Load (488 ± 203; *p* = 0.001; ES = 0.31, small effect), RHIE (9 ± 8; *p* = 0.001; ES = 0.75, moderate effect) and number of accelerations (4 ± 5; *p* = 0.001; ES = 0.49, small effect). In contrast, forwards had higher Player Load (slow) (186 ± 86; *p* = 0.52; ES = 0.06, trivial effect) than backline players.

Table 2 shows the match running demands of rugby union players from 2016 to 2018 Varsity Cup tournaments. In 2016, forwards ran the highest total distance (4370 ± 2062 m), with the players running less in 2017 (4145 ± 1902 m; ES = 0.11, trivial effect) and 2018 (3821 ± 1937 m; ES = 0.27, small effect). The backs ran larger total distances in 2017 (5284 ± 1856 m) compared to 2016 (5092 ± 2293 m; ES = 0.09, trivial effect) and 2018 (4952 ± 2275 m; ES = 0.16, trivial effect). In 2017, the forwards covered more distance in high-speed running (268 ± 176 m) and very high-speed running (87 ± 92 m) than in 2018 (237 ± 185 m; 70 ± 83 m), although trivial effects were observed.

## Discussion

The aim of this study was to analyse the match loads of rugby union players between the 2016 and 2018 Varsity Cup competitions. The findings showed that backline players covered total distances of 5105 m while forwards ran 4097 m during rugby matches. The reason why forwards covered less distance is because of the game dynamics as they are more involved in set pieces and collisions. However, the present results are lower than those of Yamamoto et al. ^[17]^ who reported that backline players and forwards covered total distances of 6392 m and 5731 m, respectively. The low total distances observed in the current study may be attributed to the fact that the sample consisted of semi-professional rugby union players from the university competition, while that of Yamamoto et al. ^[17]^ included professional players from the Japanese domestic league teams. This supports Gabbett et al.,^[14]^ who highlighted that the professional rugby league places considerable physical demands on the aerobic energy system. Therefore, the level of competitiveness may differ within teams of elite and less elite players, as well as teams playing in different countries.^[3–8, 14]^ The greater physical demands on backline players in the present study demonstrates that conditioning drills can then be tailored to the specific playing position match demands. ^[11]^

Backline players covered longer distances at very high-speed levels and with sprinting than forwards, by a large proportion. These findings are in line with those of Austin et al. ^[5]^ and Lacome et al. ^[13]^ respectively who reported that forwards run significantly lower total distances during a match. A plausible reason for this finding could be that backline players cover larger distances in matches because they generally run from a deeper position in field than their forward counterparts, thus creating more space for the backline players to gain speed for their runs with the ball in hand. ^[13]^ Consistent with previous research, ^[11]^ the present study suggests that forwards should be prescribed more low intensity activities than backline players but should complete prescribed high speed and sprinting distances.

This study also found that backline players had a significantly higher total Player Load than forwards. A possible explanation for this may be that backs are attributable to both being tackled and having short bursts of changes of direction during matches.^[10]^ It should be noted, however, that the magnitude of the difference in total Player Load was small between backline players and forwards, demonstrating that there are minor variations in the physical demands of rugby matches at university level. The current study also indicated that forwards had greater Player Load (slow) than backline players. Therefore, it seems that both backline players and forwards accumulate similar loads from low velocity activities such as tackles and physical collisions. ^[10]^ This result contrasts with previous research which found that backline players achieved a higher Player Load (slow) than forwards in New Zealand. ^[10]^ Such discrepancies could be due to different playing tactics or strategies and the physical capacities of players across countries.

Backline players recorded a significantly higher number of accelerations than the forwards. Yamamoto et al. ^[17]^ indicated that backline players are more likely to perform intense accelerations than their forward counterparts during the game. It has been previously reported by McLellan,^[18]^ that backline players are found in more space on the outer edges of the field. As a result, they need to accelerate to reach the opposition when carrying the ball, as well as having to sprint when performing kicks and chases.^[18]^ In view of the importance of acceleration in rugby union, the training programmes should consider the differences in the playing positions. Sprint training programmes for rugby players should focus on developing acceleration qualities for all playing positions, with the greater emphasis on backline players. Preferably, forwards should emphasise acceleration from a standing start, while backline players are needed to effectively change between jogging and sprinting.^[19]^

In the 2016 Varsity Cup, forwards ran their highest total distance, while running less in the 2017 and 2018 Varsity Cups respectively. When the Point of Origin law was introduced in 2016, the teams may have been uncertain how to fully utilise this law to their advantage and therefore this may be a reason as to why the forwards ran their highest distance during matches in 2016.^[20]^ A variation in this law occurred in 2017, which may have influenced the team to change their in-game tactics to use this variation to their benefit. This law was aimed at promoting attacking rugby and ball retention, and therefore players may have had increased possession and opportunities to run further during matches.^[20]^

### Limitations and future research

Although this study provided novel information on match running demands of university rugby union players, certain limitations should be noted. The current study consisted of players from one team, which limits the generalisation of findings to the whole population. Furthermore, this study did not consider a specific playing position (i.e. front row forwards, back row forwards, inside backs and outside backs), locomotive characteristics (accelerations and decelerations) and situational variables (quality of opponents and match outcome). Future studies should also combine GPS to match physical demands and technical indicators using video-based performance to provide a comprehensive reflection of the more specific running position profiles of university rugby players.

## Conclusion

This study found that backline players significantly covered greater total distances, high-speed running, very high-speed running and sprinting distance than forwards. The backline players also had higher averages on RHIE, total Player Load and number of accelerations than forwards. These results have practical implications for rugby coaches in the development and implementation of individualised training sessions according to playing position. Therefore, it is recommended that training for backline players should focus on developing aerobic capacity and sprint training sessions in a match scenario. Similarly, low speed running exercises should be recommended for forwards while completing the prescribed high-speed and sprinting distances.

## Figures and Tables

**Table 1:** Overall match running demands of rugby players according to playing position

Variable	Forwards Mean ± SD	Backs Mean ± SD	p-value	Effect Size
Total distance (m)	4097 ± 1971	5105 ± 2150	0.00*	0.49 (small)
Moderate speed running (m)	1821 ± 877	1868 ± 828	0.52	0.06 (trivial)
High-speed running (m)	262 ± 189	496 ± 258	0.00*	1.03 (moderate)
Very high-speed running (m)	85 ± 93	260 ± 136	0.00*	1.50 (large)
Sprinting distance (m)	19 ± 34	117 ± 99	0.00*	1.32 (large)
Meter per minute (m)	197 ± 85	238 ± 94	0.00*	0.46 (small)
Total Player Load	426 ± 201	488 ± 203	0.00*	0.31 (small)
Player Load (slow)	186 ± 86	181 ± 75	0.52	0.06 (trivial)
RHIE	4 ± 5	9 ± 8	0.00*	0.75 (moderate)
Number of accelerations (n)	2 ± 3	4 ± 5	0.00*	0.49 (small)

* indicates significant at p<0.05.

Total Player Load, Player Load (slow) and RHIE expressed as arbitrary units. RHIE, repeated high intensity effort.

**Table 2:** Match running demands of rugby players according to year and playing position

	2016		2017		2018	

Variable	Forwards	Backs	Forwards	Backs	Forwards	Backs
Total distance (m)	4370 ± 2062	5092 ± 2293	4145 ± 1902	5284 ± 1856	3821 ± 1937	4952 ± 2275
Moderate-speed running (m)	1776 ± 872	1737 ± 855	1911 ± 890	2026 ± 752	1774 ± 871	1845 ± 854
High-speed running (m)	285 ± 205	498 ± 274	268 ± 176	502 ± 236	237 ± 185	489 ± 265
Very high-speed running (m)	102 ± 102	257 ± 144	87 ± 92	258 ± 114	70 ± 83	264 ± 147
Sprinting distance (m)	18 ± 31	110 ± 94	22 ± 42	112 ± 78	16 ± 27	127 ± 119
Metres per minute	211 ± 94	244 ± 105	197 ± 83	246 ± 82	185 ± 78	226 ± 93
Total Player Load	419 ± 189	467 ± 211	445 ± 204	513 ± 175	414 ± 209	485 ± 219
Player Load (slow)	195 ± 82	178 ± 78	186 ± 87	187 ± 63	178 ± 89	179 ± 82
RHIE	1 ± 2	3 ± 3	2 ± 4	6 ± 6	7 ± 7	16 ± 9
Number of accelerations (n)	1 ± 1	1 ± 1	1 ± 3	3 ± 4	5 ± 4	8 ± 5

Data expressed as mean ± SD. Total Player Load, Player Load (slow) and RHIE expressed as arbitrary units. RHIE, repeated high intensity effort.
